# Multiple Roles for Chemokines in Neutrophil Biology

**DOI:** 10.3389/fimmu.2020.01259

**Published:** 2020-07-09

**Authors:** Arianna Capucetti, Francesca Albano, Raffaella Bonecchi

**Affiliations:** ^1^Department of Biomedical Sciences, Humanitas University, Pieve Emanuele, Italy; ^2^Humanitas Clinical and Research Center - IRCCS, Rozzano, Italy

**Keywords:** atypical chemokine receptors, chemokine receptors, chemokines, neutrophils, neutrophil subpopulations

## Abstract

Chemokines are recognized as the most critical mediators for selective neutrophil recruitment during inflammatory conditions. Furthermore, they are considered fundamental regulators of neutrophil mobilization from the bone marrow (BM) to the bloodstream and for their homing back at the end of their life for apoptosis and clearance. However, chemokines are also important mediators of neutrophil effector functions including oxidative burst, degranulation, neutrophil extracellular trap (NET)osis, and production of inflammatory mediators. Neutrophils have been historically considered as a homogeneous population. In recent years, several maturation stages and subsets with different phenotypic profiles and effector functions were described both in physiological and pathological conditions such as infections, autoimmunity, and cancer. The aim of this review is to give an overview of the current evidence regarding the role of chemokines and chemokine receptors in neutrophil biology, including their possible role in neutrophil maturation, differentiation, and in defining emerging neutrophil subsets.

## Introduction

Neutrophils are essential players of the innate immune response and their function is strictly dependent on their trafficking. Indeed, under homeostatic conditions they have a short half-life and a constant circulating neutrophil number has to be maintained to ensure their timely recruitment during inflammation. Neutrophils produced in the bone marrow (BM) are released in the bloodstream and, after the aging process they return to the BM, spleen, lungs, or liver for their clearance. During inflammation neutrophils extravasate quickly in the tissues and consequently there is an increased neutrophil release from the BM (1, 2).

Many studies have demonstrated that neutrophils can sense different classes of chemoattractants such as leukotrienes, anaphylatoxins, and formylated peptides that are primary activators during inflammation. Moreover, they express several chemokine receptors that finely tune their directional migration in homeostatic conditions and mediate their effector functions such as oxidative burst and neutrophil extracellular trap (NET) activation and release, once extravasated in tissues (3). The ability to respond to multiple chemokines represents a mechanism to finely control neutrophil recruitment and activation providing a first line defense (4).

It is now clear that several BM and circulating neutrophil subpopulations exist with different expression patterns of chemokine receptors (4, 5). In this review, the role of chemokines in neutrophil biology will be discussed, trying to dissect their role in neutrophil differentiation, heterogeneity, and activation.

## Chemokines Acting on Neutrophils

Neutrophils respond to a multitude of chemokines via binding to their cell-surface receptors, called chemokine receptors belonging to a family of seven-transmembrane domain G protein–coupled receptors. Chemokines are divided into four structural groups (C, CC, CXC, and CX_3_C) based on the spacing of two conserved cysteine residues at their N terminal (6).

Neutrophils are generally thought to be limited in expression of chemokine receptors, consisting predominantly of the CXC group. Indeed, neutrophils express high levels of the CXC chemokine receptors CXCR1 and CXCR2 that bind ELR^+^-CXC chemokines (containing a glutamate–leucine–arginine motif before the amino-terminal CXC motif). hCXCR2 is a promiscuous receptor binding seven different chemokines CXCL1, 2, 3, 5, 6, 7, and 8 (7). hCXCR1 is very similar to CXCR2 (78% of sequence homology) but only binds CXCL6 and CXCL8 (8).

CXCR1, CXCR2, and their ligands were also identified in the murine system but there are many differences with their human counterpart (9, 10). First, in mice there are fewer ELR^+^-CXC chemokines and the homolog of CXCL8 is missing. Its analogs in mice are CXCL1 (KC), CXCL2, and CXCL5 (LIX). Mouse CXCR1 was only recently cloned and shown to be a functional receptor for the mouse chemokines CXCL5/LIX and CXCL6/GCP-2 (11).

CXCR2 activates many G-protein–induced signaling cascades: PI3K/Akt inducing cell migration, PLC/PKC that affects cell function, and mitogen-activated protein kinase (MAPK)/p38 that promotes cell proliferation and survival (12). CXCR1 and CXCR2 signaling activates the NF-κB pathway, inducing the transcription of many cytokines among which are CXC chemokines that amplify neutrophil recruitment (13). Very interestingly, only CXCR1 activates phospholipase D (PLD), involved in radical oxygen species (ROS) generation. This difference is due to a slower rate of internalization of CXCR1 compared to CXCR2 (14, 15).

Recent results outline differential roles among ELR^+^-CXC chemokines in neutrophil extravasation and migration. CXCL2 is almost exclusively produced by neutrophils and, in addition to CXCR1 and CXCR2, it binds the atypical chemokine receptor ACKR1. ACKR1, expressed by endothelial cell (EC) junctions on post capillary venules, works as a CXCL2 presenter guiding neutrophils to extravasation sites. Otherwise, CXCL1 mediates neutrophil adhesion and intraluminal crawling on inflamed ECs and sub-EC crawling on pericytes (16).

Neutrophils express also the CXC receptor CXCR4, essential for their life cycle. BM neutrophils express high levels of CXCR4, which is mainly intracellular because of high CXCL12 production by mesenchymal cells inducing its internalization (17). The interaction between CXCR4 and CXCL12 retains a large pool of neutrophils into BM and spleen (18). This is demonstrated by CXCR4 genetic deletion in murine myeloid cells that results in depletion of the BM pool and in concomitant increase of circulating neutrophils (19). On the contrary, WHIM (warts, hypogammaglobulinemia, infections, myelokathexis) patients, who bear a gain of function mutation in CXCR4, have a chronic neutropenia for increased neutrophil BM retention (20). Circulating neutrophils express low levels of CXCR4 that is upregulated in senescent neutrophils before apoptosis, promoting their homing back to the BM and other organs for clearance (21). Studies *in vitro* demonstrated that CXCR4 is downregulated by type I cytokines such as interferon-γ (IFN-γ), IFN-α, granulocyte-macrophage colony stimulating factor (GM-CSF), and granulocyte-colony stimulating factor (G-CSF) (22).

CC chemokine receptors are barely expressed by BM and circulating neutrophils. When neutrophils are activated by IFN-γ or GM-CSF, they upregulate the expression of CCR1 and CCR3 (23, 24). CCR1 was found necessary for neutrophil recruitment in a murine model of renal immunopathology (25) together with other CC receptors (CCR2, CCR3, CCR5) (26, 27).

CCR2, expression of which was previously supposed to be restricted to monocytes, is important also for neutrophils. It induces neutrophil BM mobilization (28), accumulation in joints of rheumatoid arthritis patients (29), and recruitment to metastatic sites (30, 31). A subpopulation of neutrophils with antigen presenting function expressing CCR6 and CCR7 was also described (32–34).

Finally, neutrophils express one atypical receptor named CCRL2 that, despite being very similar in structure to chemokine receptors, does not bind chemokines. CCRL2 forms dimers with CXCR2 regulating its membrane expression and function (35).

## Role of Chemokines in Granulopoiesis

**Figure 1:**
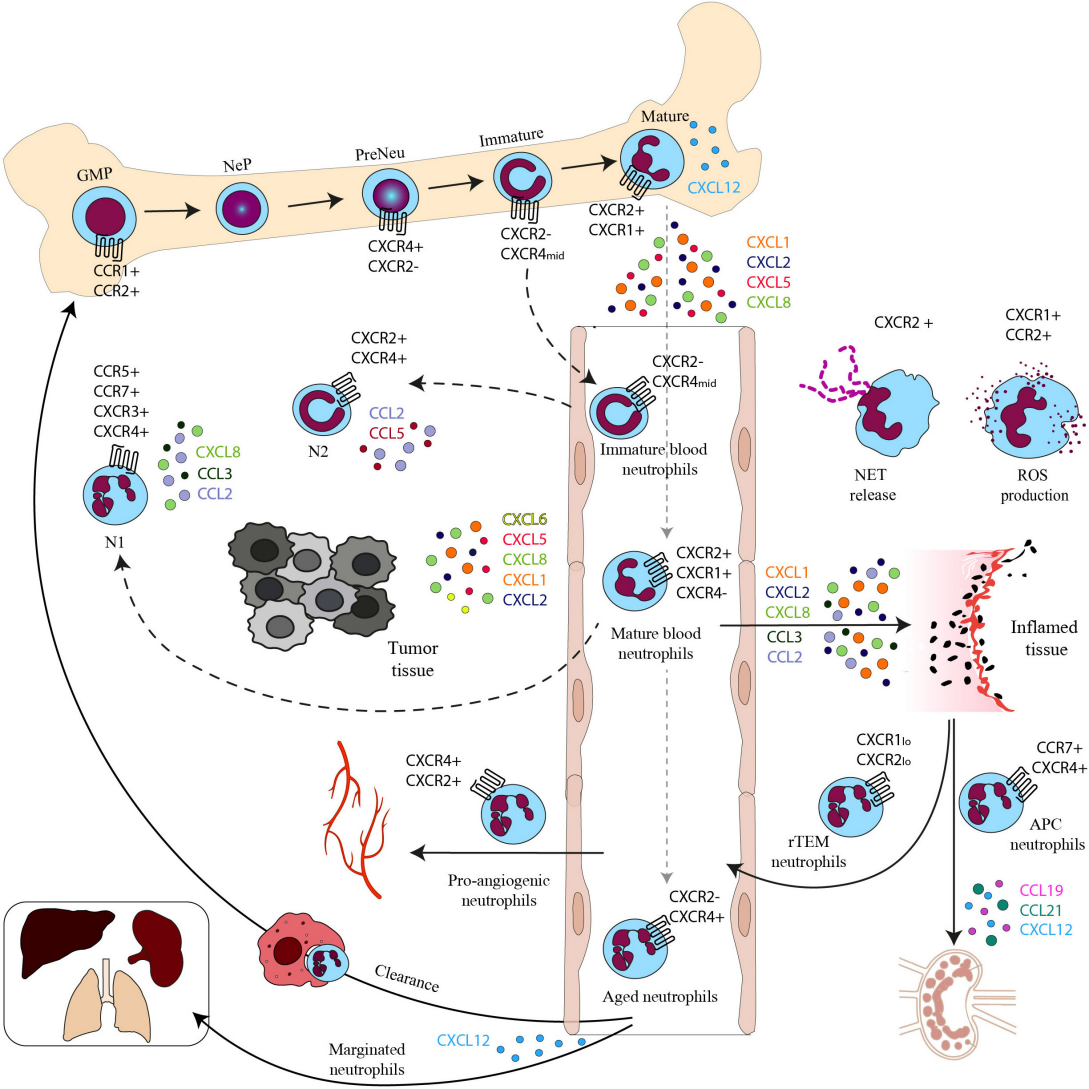
Chemokines and chemokine receptors in the neutrophil life cycle. Neutrophil progenitor proliferation is regulated by CC chemokines binding to CCR1 and CCR2. Neutrophil subpopulations are retained in the BM by the CXCL12–CXCR4 axis. Mature neutrophils are released in the bloodstream upon the upregulation of CXCR2. In the bloodstream, besides mature neutrophils, there are immature neutrophils derived from immature BM neutrophils and senescent neutrophils that upregulate CXCR4 expression and follow the CXCL12 gradient to home back to the BM or other tissues for their clearance. CXCR4 can also drive senescent neutrophils to the lungs, spleen, and liver where they reside as a marginated pool. In the bloodstream there are also rTEM with low levels of CXCR1 and 2. In inflamed tissues activated neutrophils produce ROS on stimulation of CXCR1 and CCR2 and release NETs after engagement of CXCR2. Neutrophils with APC function migrate to draining LNs by the CCR7–CCL19/21 or the CXCL12–CXCR4 axis and proangiogenic neutrophils express high levels of CXCR4 that allows their migration to hypoxic areas. Mature and immature neutrophils migrate in the tumor, where they are referred to as N1 and N2 tumor associated neutrophils (TAN).

Neutrophil maturation follows a multistep process called granulopoiesis. The most immature progenitor, the hematopoietic stem cell (HSC), gives rise to multipotent progenitors, the common myeloid progenitors (CMPs) that stimulated with G-CSF give rise to granulocyte-macrophage progenitors (GMPs). In the classical granulopoiesis model, downstream of GMPs there are neutrophil committed progenitors called promyelocytes and myelocytes (36–39). These immature proliferating progenitors are now referred as neutrophil progenitors (NePs) and neutrophil precursors (preNeu) (40); they have been transcriptionally defined and can be identified by fluorescence-activated cell sorting (FACS) analysis (Table 1). These unipotent progenitors differentiate into non-proliferating immature neutrophils (previously called metamyelocytes and banded neutrophils) and mature neutrophils (41) (Figure 1).

**Table 1:** Expression signature of neutrophil progenitors and subpopulations.

	**Mouse**	**Human**
HSC	Lin^−^, CD117^+^, Sca-1^+^, CD34^+^, CXCR4^+^	Lin^−^, CD34^+^, CD38^−^, CD45RA^−^, CXCR4^+^
CMP	Lin^−^, CD117^+^, Sca-1^−^, CD34^+^, CXCR4^+^, CCR1^+^, CCR2^+^	Lin^−^, CD34^+^, CD38^+^, CD45RA^−^, CXCR4^+^, CCR1^+^, CCR2^+^
GMP	Lin^−^, CD117^+^, Sca-1^−^, CD34^+^, CD16/32^+^, CXCR4^+^, CCR1^+^	Lin^−^, CD34^+^, CD38^+^, CD45RA^+^, CXCR4^+^, CCR1^+^
NeP	Lin^−^, CD117^+^, Ly6A/E^−^, Siglec F^−^, FcεRIα^−^, CD16/32^+^, Ly6B^+^, CD11a^+^, CD162^lo^, CD48^lo^, Ly6C^lo^, CD115^−^, Ly6G^−^, CXCR4^+^	Lin^−^, CD117^+^, CD66b^+^, CD38^hi^, CXCR4^+^
preNeu	Lin^−^, CD117^+^, CD115^−^, Siglec-F^−^, Gr1^+^, CD11b^+^, Ly6G^lo^, CXCR2^−^, CXCR4^+^	Lin^−^, CD117^−^, Siglec8^−^, CD15^+^, CD34^−^, CD66b^hi^, CD49d^+^, CD101^−^, CXCR2^−^, CXCR4^+^
Immature neutrophil	Lin^−^ CD117^−^ CD115^−^, Siglec-F^−^, Gr1^+^, CD11b^+^, Ly6G^lo/+^, CXCR2^−^, CXCR4^MID^	Lin^−^, CD66b^+^, CD15^+^, CD33^mid^ CD101^+^, CD10^−^, CD16^lo/+^, CXCR2^−^, CXCR4^−^
Mature neutrophil	Lin^−^, CD115^−^, CD11b^+^, Ly6G^+^, CXCR2^+^, CXCR4^−^	Lin-, CD66b^+^, CD15^+^, CD33^mid^, CD101^+^, CD10^+^, CD16^hi^, CXCR2^+^, CXCR4^−^
Aged neutrophil	CD11b^+^, CD16/32^+^,CD62L^lo^, CXCR2^LO^, CXCR4^HI^	CD11b^+^, CD16^hi^, CD62L^lo^, CD10^+^, CXCR2^LO^, CXCR4^HI^

The chemokine system is involved in several aspects of myelopoiesis and granulopoiesis. CXCL12 is constitutively produced by BM stromal cells and provides a retention signal for CXCR4-positive neutrophil committed progenitors and immature neutrophils. G-CSF mobilizes neutrophils through the cleavage of CXCL12 and CXCR4 (42). Beyond BM retention, it is not known if CXCR4 modulate proliferation of NePs as in the case of HSC (43).

CXCR2 signaling, interacting antagonistically with CXCR4, represents a second chemokine axis to regulate neutrophil release from the BM (44). The mobilization of neutrophils from the BM to the blood is determined by the downregulation of CXCR4 and subsequent upregulation of CXCR2 receptor both in humans and in mice (17, 45).

Other chemokines and chemokine receptors have a role in the process of granulopoiesis and neutrophil release from BM. CCL3 induces the proliferation of CCR1-positive myeloid progenitors even if the *in vivo* relevance of this effect is not evident because CCR1 KO mice do not show significant differences in CMP and GMP proliferation compared to WT (46). CCR2 is expressed by CMPs and exerts a negative control on myelopoiesis (47). In addition, CCR2 mediates mobilization from BM to peripheral blood of myeloid populations such as monocytes and neutrophils (48).

Granulopoiesis is also affected by atypical chemokine receptors. ACKR1, expressed by BM nucleated erythroid cells (49, 50), and ACKR2, expressed by hematopoietic progenitors, control neutrophil differentiation (31). However, it is not known the mechanism by which their control of the chemokine system affects neutrophil differentiation.

Thus, in homeostasis the chemokine receptors CXCR4 and CXCR2 play an essential role in controlling neutrophil retention and release from the BM. CXCR4 and CC chemokine receptors are expressed by neutrophil progenitors, but further research is needed to better understand their role in granulopoiesis.

## Role of Chemokines in Neutrophil Heterogeneity

Despite the previous belief that differentiated neutrophils were a homogeneous population, the existence of different circulating subsets was demonstrated in varied health and disease contexts, both in mice and humans (51) (Figure 1). A consensus on the phenotype of these subpopulations is still missing and under steady-state conditions heterogeneity may arise mainly from the aging process of circulating neutrophils (52). Indeed, neutrophils oscillate in a circadian manner in numbers, morphology, and phenotype (53, 54). This process is regulated by gut microbiota (55) and is controlled by neutrophils themselves through the circadian expression of the transcription factor Bmal1 that controls the production of CXCL2. In turn, CXCL2 acting on CXCR2 induces neutrophil aging (56).

During inflammatory conditions, increased levels of a neutrophil circulating population that shared characteristics with BM immature neutrophils was described both in mice and humans. These cells express low levels of CD16 and are CD10^−^ (57–59). The functional properties of this subset are still controversial, they were described having either immunosuppressive activity (60) or promoting T-cell survival and proliferation (57).

Other circulating neutrophils subpopulations were described: olfactomedin 4 (OLFM4)-positive neutrophils in healthy donors (61), T-cell receptor (TCR)–based variable immunoreceptor neutrophils (62), and CD177^+^ neutrophils during inflammatory diseases both in mice (63) and humans (64).

In addition, a reverse transendothelial migrating neutrophil subset (rTEM) was described in a murine model of sterile injury (65). These neutrophils are CD54^hi^ and, in order to reverse transmigrate into vasculature, downregulate CXCR1. Concomitantly, they upregulate CXCR4 to go into the lungs, before being cleared in BM (66). This subset represents a phenotypically and functionally distinct population different from circulating neutrophils (CD54^lo^ CXCR1^hi^) and express vascular endothelial growth factor receptor (VEGFR) 1, indicating a possible role in angiogenesis (67, 68). Similar cells, with increased levels of CD54 and CD18 and downregulation of CD62L and CXCR1 and 2, were found in patients with chronic inflammatory diseases, suggesting a role of rTME neutrophils in the persistence of inflammation (67). Moreover, around 1% of circulating neutrophils after ischemia-reperfusion were found to be CD54^hi^ and producing ROS into lungs (65). On the contrary, neutrophils that migrate away from the inflammation site in interstitial tissues are called reverse interstitial migration (rIM) neutrophils and are supposed to contribute to the resolution of inflammation. The role of chemokine receptors in this process is still not clear (69).

Finally, in circulation it is possible to identify aged or senescent neutrophils (54, 70, 71). *Ex vivo* aging experiments have shown that neutrophils kept in culture downregulate the expression of CXCR2 (44) and re-express CXCR4 in a time-dependent way (22), suggesting a preferentially homing of senescent cells to the BM in response to CXCL12 (21). In mice aged neutrophils display circadian oscillations and, in addition to high levels of CXCR4, are characterized by an increased surface expression of CCR5 and decreased expression of CD62L (53, 72). CXCR4 upregulation seems involved not only in guiding neutrophils back to the BM but also in their migration within the marrow tissue in order to be engulfed with greater efficacy by macrophages (17, 19, 53, 54, 72). CCR5 was reported to work as a chemokine scavenger promoting the resolution of the inflammatory response (73). Aged neutrophils were found in lungs, where pulmonary vasculature expresses CXCL12, and this could either supply the pool of circulating neutrophils or respond to injury (45, 68).

New data from single cell sequencing of murine circulating neutrophils confirm the presence of three transcriptionally different neutrophil subpopulations. The first expresses high levels of inflammatory genes and the highest levels of CXCR2 arising mainly from BM mature neutrophils. The second expresses interferon-stimulated genes and derives from BM immature neutrophils. Both populations mature in an aged subset CXCR4 positive with high phagocytic activity and still highly transcriptionally functional (41). The correlation of these subpopulations of neutrophils with the others described in the foregoing is still missing. In addition, the role of chemokines in the mobilization and function of these neutrophil subpopulations is not known. Of relevance, at least in mice, mobilization of immature neutrophils could be CXCR2 independent because they are referred to as CXCR2 negative (44).

Finally, neutrophil heterogeneity has been described in tumors where tumor-associated neutrophils (TANs) can exist in two different functional states: N1 proinflammatory and antitumoral subset and an antiinflammatory tumor promoting N2 population, distinguished for the expression of adhesion molecules, cytokines and inflammatory mediators, chemokines, and chemokine receptors (4, 74). N1 phenotype has been associated with IFN-β polarization both in mice and humans. These cells have an activated phenotype (CD62L^lo^ CD54^+^); express the chemokine receptors CCR5, CCR7, CXCR3, and CXCR4; and produce the proinflammatory chemokines and cytokines: CCL2, CXCL8, CCL3, and interleukin-6 (IL-6). Moreover, this subset has been associated with stimulation of T-cell responses and ROS production (4, 75, 76). In contrast, N2 neutrophils are induced by transforming growth factor- β (TGF-β) stimulation. Protumoral N2 neutrophils display high levels of CXCR4, VEGF, and matrix metalloproteinase 9 (MMP-9) (77), and produce high levels of CCL2, CCL5, neutrophil elastase (NE), cathepsin G (CG), and arginase 1 (78–80).

Therefore, results obtained in preclinical mouse models and in humans suggest that the interplay between CXCR2 and CXCR4 dictates not only BM neutrophil mobilization and retention but also neutrophil diversity in homeostasis. CXCR2 signaling promotes neutrophil aging and CXCR4 guides their homing back to the BM. Furthermore, diversity of tissue infiltrating neutrophils is also associated with a distinct pattern of chemokine receptors; in particular N1 neutrophils express inflammatory CC chemokine receptors important for their effector functions (see later).

## Role of Chemokines in Neutrophil Effector Functions

Neutrophils, once recruited to sites of infection, recognize and phagocytize microbes and then kill pathogens with different cytotoxic mechanisms. These include the production of ROS, the release of antimicrobial peptides, and the expulsion of their nuclear contents to form NETs. Moreover, neutrophils can also shape the immune response interacting with adaptive immune cells (1, 68) (Figure 1).

The chemokine system, fundamental for selective neutrophil recruitment in the tissues, also has an important role in the regulation of the effector functions of neutrophils. Engagement of both CXCR1 and CXCR2 induces neutrophil activation but the two receptors have distinct and non-redundant roles in inflammation and infection. Studies with knockout mice proved the importance of mCXCR2 in inflammatory diseases related to neutrophil infiltration and activation (30, 81). On the contrary, mCXCR1 appears dispensable for neutrophil transmigration while necessary for ROS production in *Pseudomonas aeruginosa* and degranulation in *Candida albicans* infections (82, 83). The fundamental role of CXCR1 in fighting infections is further confirmed in humans carrying a genetic variant of CXCR1 (CXCR1–T276) that have increased bacterial infections. Neutrophils taken from these individuals have impaired degranulation and fungal killing ability (83). On the contrary, ROS production induced by the CXCL8–CXCR2 axis on circulating neutrophils has a regulatory function. Indeed, it limits the rolling capability of neutrophils in an autocrine manner by inducing the shedding of CD62L (83).

In inflammatory conditions and after extravasation, neutrophils completely change their chemokine receptor repertoire. They downregulate CXCR2 levels and upregulate inflammatory CC receptors CCR1, CCR2, and CCR5. These receptors activate neutrophil phagocytic activity and ROS production (26, 27, 84). In murine models of breast lung metastasis, CCR2 expression on neutrophils promotes ROS production that kills cancer cells (31, 85).

Release of NETs is induced by CXCR2 activation via Src, extracellular signal-regulated kinase (ERK), and p38/MAPK signaling (86). The CXCL1–CXCR2 axis has been associated to NET formation and neutrophil degranulation in a model of deep vein thrombosis in mice (87) and in circulating and airway mucosal neutrophils of chronic obstructive pulmonary disease (COPD) patients. The use of CXCR2 antagonist in these patients had significantly improved their lung function, even if a direct effect in NETs inhibition was not proved (88). Clinical trials are ongoing for the use of CXCR2 inhibitors in COPD patients (Table 2). NETs role in cancer still remains controversial; indeed, they have been associated with both pro- and antitumoral functions (89). In diffuse large B-cell lymphoma, CXCL8-induced NETs promote tumor progression and blocking the CXCL8–CXCR2 axis delays cancer progression in preclinical models (90). CXCR1 and CXCR2 inhibitors show encouraging results in tumor preclinical models when they are used in combination with chemotherapy and checkpoint inhibitors and are now in use in several clinical trials (Table 2) (91).

**Table 2:** Clinical trials with CXCR1 and CXCR2 inhibitors.

**Target**	**Inhibitor**	**Pathology**	**Clinical trials**	**Results**
CXCR2	AZD5069	Asthma and bronchiectasis	NCT01704495 NCT01255592	Reduced neutrophils in sputum and lung tissue; no improvement in clinical outcomes
		Advanced solid and metastatic tumors (head and neck carcinoma, prostate cancer, pancreatic cancer)	NCT02499328 NCT03177187 NCT02583477	Not available
	Danirixin (GSK1325756)	COPD	NCT02130193 NCT03250689	Improvements in respiratory symptoms; reduced NET formation
		Viral disease (influenza)	NCT02469298	Termination for emergence of severe adverse events (cardiac failure and respiratory disease)
	SB-656933	Ulcerative colitis,	NCT00748410	No clinical benefit
		Cystic fibrosis	NCT00903201	Improved inflammatory markers in patients' sputum; no change in lung function
CXCR1 and CXCR2	Reparixin	Liver, lung, and kidney transplantation	NCT03031470 NCT00224406 NCT00248040	Attenuated inflammatory reaction and reduced tissue damage
		Islet transplantation in diabetes mellitus type 1	NCT01817959	No improvement in islet inflammation-mediated damage
		Metastatic breast cancer	NCT02370238 NCT02001974	Not available
	Navarixin (SCH 527123, MK-7123)	COPD	NCT01006616	Improved clinical outcomes
		Advanced/metastatic solid tumors (in combination with pembrolizumab)	NCT03473925	Not available
		Psoriasis	NCT00684593	No clinical benefit

Chemokines acting on neutrophils can also regulate angiogenesis in a direct and indirect way. CXCL1 induces VEGF-A production by neutrophils (92), and neutrophils with an aged-like profile (VEGFR1^+^, CD49d^+^, and CXCR4^+^), recruited to hypoxic areas where CXCL12 is produced, promote angiogenesis by release of MMP9 that cleaves VEGF-A stored in the matrix (93–95).

Of relevance, neutrophils recruited at an inflammatory site orchestrate and polarize the immune response, producing many chemokines. ELR^+^CXC and inflammatory CC chemokines amplify innate immune cell recruitment. Neutrophils can also promote the recruitment of lymphocytes producing the Th1 chemokines CXCL9, 10, and 11 and the B-cell attracting chemokine CXCL13. On the contrary, TANs exert their immunosuppressive function by producing the Treg attracting chemokine CCL17 (96). Moreover, a subset of activated neutrophils expressing CCR7 and CXCR4 can migrate to lymph nodes (LNs) and act as antigen presenting cells (APC) (97, 98).

According to these results, CXCR2 expression on neutrophils, besides being fundamental for extravasation, induces NET release, while CXCR1 together with CC chemokine receptors CCR1, CCR2, and CCR5 acquired by infiltrated neutrophils promote degranulation and ROS production. Chemokines produced by neutrophils in inflamed tissues amplify and polarize the immune response, and the expression of CCR7 by activated neutrophils promotes their migration to LN, where they can directly act as APC.

## Concluding Remarks

Chemokines and their receptors play multiple and non-overlapping roles in the life span of a neutrophil. CXCR4 has a central role for BM retention of immature neutrophils and BM homing of aged neutrophils. On the contrary, CXCR2 induces neutrophil mobilization from the BM to the bloodstream and has a critical role in neutrophil extravasation, NET release, and the aging process. CXCR1 together with CCR1, CCR2, and CCR5 are important for degranulation and ROS production after extravasation. The production of inflammatory chemokines by neutrophils at an inflammatory site amplifies and polarizes the immune response, and CCR7 and CXCR4 expression guides neutrophil migration to draining LNs for antigen presentation.

Clinical trials using CXCR1 and CXCR2 inhibitors revealed that they are successful in treating patients with chronic diseases (e.g., COPD), whereas their use can be detrimental in patients with viral infection. Therefore, a better understanding of the role of chemokines not only in neutrophil migration but also in diversity, effector functions, and regulation of the immune response is required to develop successful therapeutic strategies.

## Author Contributions

All authors have equally contributed to this review by writing and critically evaluating the literature.

## Conflict of Interest

The authors declare that the research was conducted in the absence of any commercial or financial relationships that could be construed as a potential conflict of interest.
